# Complete Genome Sequence of *Escherichia coli* 81009, a Representative of the Sequence Type 131 C1-M27 Clade with a Multidrug-Resistant Phenotype

**DOI:** 10.1128/genomeA.00056-18

**Published:** 2018-02-22

**Authors:** Michele Mutti, Ágnes Sonnevend, Tibor Pál, Sini Junttila, Heinz Ekker, Bence Galik, Attila Gyenesei, Gábor Nagy, Eszter Nagy, Valéria Szijártó

**Affiliations:** a Arsanis Biosciences GmbH, Vienna, Austria; b Department of Medical Microbiology and Immunology, United Arab Emirates University, Al Ain, United Arab Emirates; c BioComp, Bioinformatics & Scientific Computing, Vienna Biocenter Core Facilities GmbH, Vienna, Austria; d Next Generation Sequencing, Vienna Biocenter Core Facilities GmbH, Vienna, Austria

## Abstract

The sequence type 131 (ST131)-*H*30 clone is responsible for a significant proportion of multidrug-resistant extraintestinal *Escherichia coli* infections. Recently, the C1-M27 clade of ST131-*H*30, associated with *bla*_CTX-M-27_, has emerged. The complete genome sequence of *E. coli* isolate 81009 belonging to this clone, previously used during the development of ST131-specific monoclonal antibodies, is reported here.

## GENOME ANNOUNCEMENT

*Escherichia coli* strain 81009 was isolated from the urine of a 76-year-old male patient treated at Tawam Hospital, Al Ain, United Arab Emirates (1), and has recently been deposited at the Polish Collection of Microorganisms (PCM-2857). This strain belongs to sequence type 131 (ST131) and expresses the O25b O-antigen (2). *E. coli* 81009 is resistant to monobactams, cephalosporins, streptomycin, and fluoroquinolones and carries the *bla*_CTX-M-27_ allele. The recently emerging ST131-*H*30 isolates expressing CTX-M-27 are considered to form a new clade (C1-M27) within this clone (3, 4).

Genomic DNA of *E. coli* 81009 was extracted with the Genomic-tip 100/G kit and Genome buffer set (Qiagen) from bacteria grown in LB supplemented with 100 μg/ml ampicillin to mid-log phase (optical density at 600 nm [OD_600_], ∼0.5). The genomic material was sequenced on a PacBio RS II platform (Pacific Biosciences, USA) and with the Illumina HiSeq 2500 platform with a paired-end (PE) 125-bp read length (Illumina, The Netherlands).

Hierarchical Genome Assembly Process 4 (HGAP4) (5) was used for *de novo* assembly of the PacBio reads, and Illumina short reads were aligned to the *de novo* assembled genome with the Burrows-Wheeler Alignment tool (BWA) (6), followed by indel realignment and base quality recalibration with Genome Analysis Toolkit (GATK) (7). Protein-coding genes were called with Prodigal within the Prokka annotation pipeline (8). Trusted protein sequences for the annotation step were obtained from *E. coli* ST131 EC958 (9). Additional coding and noncoding RNA genes, as well as signal peptides, were predicted by RNAmmer, ARAGORN, SignalP, and Infernal within the Prokka annotation pipeline. The PlasmidFinder version 1.3 server (10) and ResFinder version 2.1 server (11) were used to identify plasmid *repA* genes and antibiotic resistance determinants.

*E. coli* 81009 has a circular chromosome of 5,012,084 bp and one plasmid (pEC-81009) with a length of 135,720 bp. The chromosome has a GC content of 50.8% and contains 4,987 predicted genes encoding 4,668 proteins and 319 RNAs (including 87 tRNAs, 22 rRNAs, and 1 transfer-messenger RNAs [tmRNAs]). A putative function was assigned to 91.7% of the protein-coding genes. The extraintestinal pathogen designation of *E. coli* 81009 was supported by an array of virulence determinants identified *in silico* (12), including those involved in adhesion (*upaB*, *crl*, *csgA*, *fimH*, and *iha*), biofilm formation (*bscA*, *agn43*, and F9 fimbriae), survival in body fluids (*iss* and *gad*), and toxin production (*senB* and *sat*). *E. coli* 81009 was also shown to carry the *H*30 allele of the *fimH* gene. Additionally, an 11.8-kb region (M27PP1) specific to the C1-M27 clade of the *H*30 subclone (4) was identified, confirming that strain 81009 belongs to the C1-M27 clade.

Plasmid pEC-81009 was found to belong to the IncFII-FIA-FIB group by *in silico* replicon typing (10). Among the 160 predicted genes carried on this plasmid, toxin/antitoxin systems (Phd/Doc, CcdBA, and Kid/Kis) and genes encoding resistance to aminoglycosides (*aadA5*, *strA*, and *strB*), sulfonamide (*sul1* and *sul2*), tetracycline [*tet*(A)], macrolides (*mphA*), trimethoprim (*dfrA17*), and the extended-spectrum β-lactamase (ESBL) gene *bla*_CTX-M-27_ were identified.

The availability of *E. coli* 81009 in a public repository and its well-characterized phenotype and genotype make it a suitable model organism for studying the ST131 C1-M27 clade.

### Accession number(s).

The complete sequences of the chromosome and plasmid of *E. coli* 81009 have been deposited in GenBank under accession numbers CP021179 and CP021180, respectively.
